# Preteen children’s health related quality of life in Sweden: changes over time and disparities between different sociodemographic groups

**DOI:** 10.1186/s12889-019-6429-6

**Published:** 2019-01-31

**Authors:** Mazen Baroudi, Solveig Petersen, Fredinah Namatovu, Annelie Carlsson, Anneli Ivarsson, Fredrik Norström

**Affiliations:** 1 Department of Epidemiology and Global Health, Umeå University, Umeå, Sweden; 2 Department of Historical, Philosophical and Religious Studies, Umeå University, Umeå, Sweden; 3 Department of Pediatrics, Clinical Sciences, Skåne University Hospital, Lund University, Lund, Sweden

**Keywords:** Preteen children health, Health inequity, Quality of life, HRQoL, Sociodemographic disparities

## Abstract

**Background:**

Assessing disparities in health-related quality of Life (HRQoL) is important as a part of health-related disparities in the society. The aim of this study was to explore HRQoL among 12-year-olds in Sweden in terms of differences between years 2005 and 2009 and disparities related to sociodemographic background.

**Methods:**

During the school years 2005 and 2009, a total of 18,325 sixth grade students in Sweden were invited to a celiac disease screening study; 13,279 agreed to participate. Jointly with the celiac screening, the children answered a questionnaire that included EuroQol 5 Dimensions-youth (EQ-5D-Y) and their parents responded to separate questionnaires about their own and their child’s country of birth, family structure, their employment status, occupation, and education. In total 11,009 child-parent questionnaires were collected. Logistic regression was used to study differences in HRQoL between 2005 and 2009, and between various sociodemographic subgroups.

**Results:**

Compared with 2005, children in 2009 reported more pain (OR: 1.20, 95% CI: 1.1–1.3) and more mood problems (OR: 1.35, 95% CI: 1.2–1.5). In general, girls reported more pain and mood problems and had more disparities than boys. There were no significant differences based on parents’ occupation, however, children of parents with low or medium education levels reported less “mood problems” than those of parents with high education levels (OR: 0.65, 95% CI: 0.46–0.92) and (OR: 0.84, 95% CI: 0.73–0.96), respectively. A slight variation was seen in HRQoL between children with different migration background. Girls living in small municipalities reported more pain (OR: 1.51, 95% CI: 1.14–2.01), and problems performing usual activities (OR: 3.77, 95% CI: 2.08–6.84), compared to girls living in large municipalities. In addition, children living with two parents had less mood problems than children living in other family constellations.

**Conclusion:**

More children reported pain and mood problems in 2009 compared with 2005. To study future trends, health outcomes among children in Sweden should continue to be reported periodically. More efforts should be invested to increase the awareness of health-related disparities as highlighted in this study especially for girls living in small municipalities and children of parents with high education level.

**Electronic supplementary material:**

The online version of this article (10.1186/s12889-019-6429-6) contains supplementary material, which is available to authorized users.

## Background

There is a growing interest in health-related quality of life (HRQoL) measures as a tool to evaluate health related functioning and wellbeing in a population. This measure has been proved valuable when exploring healthcare needs, evaluating health system performance and as a basis for resource allocation [1, 2]. More recently, HRQoL has been used to evaluate inequities between different groups in the society [3–6].

Health inequity refers to systematically exposing disadvantaged social groups to further disadvantages with regards to their health. On the other hand, health inequality is a normative free concept that refers to differences in health that are neither systematic nor unfair. However, when measuring differences in health between various social group, health inequalities may indirectly reflect inequities as it shows health advantages to already socially advantaged groups [7, 8]. To identify, monitor and act upon health inequities, the PROGRESS framework suggests that various factors should be taken into consideration. PROGRESS stands for; Place of residence, Race/ethnicity/culture/language, Occupation, Gender/sex, Religion, Education, Socioeconomic status and Social capital [9]. This framework has been adapted for evaluation of health related inequities by for instance the PRISMA-Equity Bellagio group [10].

Few studies have addressed changes over time in health and HRQoL among younger school-aged children in Sweden, and even fewer have captured the view of the child [11]. Given the low to moderate parent-child agreement in reports of health-related issues in general populations, self-reported measures are advised [12, 13]. The available literature on parent and child reported aspect of HRQoL e.g. psychosomatic and depressive symptoms, are conflicting. Some studies show no changes over time of such symptoms among 11-year-olds while others show increasing HRQoL problems in the preteen children [14, 15]. Thus, there is a need to further investigate changes over time in self-reported HRQoL among preteen children.

Assessing sociodemographic disparities in child HRQoL is important, as such disparities is unjust and might affect health later in life [16]. Previous studies have reported associations between subjective health outcomes and some sociodemographic characteristics [17–23]. Age and sex appear to play a role in HRQoL disparities in Sweden with better HRQoL among younger than older children and among boys compared to girls [17, 18]. Family structure may also play a role for children’s HRQoL. A Swedish study has shown that children who live with two parents, or in a joint custody, reported better HRQoL than those living mainly with one parent [19]. Regarding migration status, results are contradictory; some Swedish studies suggest that adolescents with a migrant background experience more health complaints and worse HRQoL than native Swedish adolescents [20, 21], while others have shown that migrant adolescents experience less or as much ill-health as Swedish adolescents [22]. The role of parents’ education and occupation are not well studied in Sweden and the international literature is conflicting. Some studies indicate that higher education may have a positive impact on preteens’ HRQoL [23]. Others find no overall variation in relation to parents’ education but indicate differences between countries [3, 5]. The major part of the literature on social disparities in child HRQoL and related health outcomes targets adolescents only [21], or a wide age group with no possibility to obtain specific information about disparities among younger children [3, 5, 22]. However, sociodemographic disparities in health and HRQoL could differ between preteen children and teenagers [19, 23]. Thus, there is a need to further investigate the social distribution of child health related outcomes in different cultures, and a particular need of studies targeting sociodemographic disparities among preteen children.

The aim of our study was to explore HRQoL among 12-year-olds in Sweden with respect to differences between the years 2005 and 2009, and disparities related to sociodemographic characteristics.

## Methods

### Study population

A repeated cross-sectional study was conducted during the school years starting August 2005 and 2009 (onwards named 2005 and 2009). The main objective of this school-based study, the ETICS study (Exploring The Iceberg of Celiac in Sweden), was the screening of celiac disease. The study comprised two cohorts of children in 6th grade at five study sites in Sweden: Lund/Malmö, Växjö, Norrköping, Norrtälje and Umeå [24].

Out of the 18,325 invited children, 13,279 agreed to participate in the study. As part of the study, children answered a questionnaire at school together with the screening while parents answered a questionnaire at home. In total, 12,658 of the children (95%) and 11,239 of the parents (85%) filled in the questionnaire. In this study, we included the 11,009 children (60% of the invited) with responses from both themselves and their parents; 6449 from the first cohort and 4560 from the second cohort. There were 1419 children’s questionnaires without parents’ responses where only information about child-reported HRQoL dimensions are available. Children were asked about their HRQoL, while parents responded to questions about their own and their child’s country of birth, family structure, their employment status, occupation, and education.

### Measures

HRQoL in the general population can be measured by a variety of instruments. Of these, the EuroQol 5 Dimensions (EQ-5D) captures the HRQoL of adults and EQ-5D-Y (Youth) is a child-friendly version of the adult instrument. The EQ-5D-Y has been tested in Sweden and international studies present evidence of its feasibility, reliability and validity. These studies support the discriminative validity of the tool i.e. its ability to differentiate between children belonging to different sociodemographic groups [25–27].

Five dimensions of HRQoL were measured by the EQ-5D-Y [28]. These dimensions correspond to *mobility* in terms of ability to walk; looking after one-self (*self-care*) in terms of independence in daily personal care, especially regarding washing and dressing; *performing usual activities* such as going to school, family and spare-time activities, hobbies, sports and playing; having pain or discomfort (*pain*), and finally, feeling worried, sad or unhappy (*mood problems*). Response alternatives correspond to no, some or severe current problems. In the analysis, ‘some’ and ‘severe’ problems were combined as one category.

The inclusion of potential explanatory factors of HRQoL disparities was guided and ordered by the PROGRESS framework [9]*.*

*Place of residence,* the 23 included municipalities were categorized according to population size. As suggested by the Swedish Central Bureau of Statistics [29], large municipalities was defined by areas with more than 50,000 inhabitants, middle size municipalities by areas with 10,000 to 50,000 inhabitants, and small municipalities by areas with less than 10,000 inhabitants.

*Migration status* was categorized into four groups based on child and parents’ country of birth: Swedish origin, i.e. the child and both parents were born in Sweden. One foreign parent, i.e. the child and one parent were born in Sweden. Foreign parents, i.e. the child was born in Sweden but both parents were born outside Sweden. Foreign origin, i.e. the child and both parents were born outside Sweden. This approach is in accordance with Hjern et al. [21].

*Parents’ occupation* was measured by the socioeconomic index (SEI) which is based on the Swedish Socioeconomic Classification (SEI-1982) [30]. This index captures the highest occupation and employment status in the household. Household SEI was classified into skilled non-manual workers including executives and self-employed professionals (SEI-codes: 54–57 and 60–87), unskilled non-manual worker including assistants and intermediate non-manual workers (SEI-codes: 33–46), skilled manual worker (SEI-codes: 21–22), unskilled manual worker (SEI-codes: 11–12), and students (SEI-codes 01–03). The SEI of the currently employed parent was used if the other parent was unemployed. The household occupation and employment status was defined as student if both parents were students or one parent was a student and the other unemployed for more than six months.

*Parents’ education level* was categorized into low, medium and high education levels based on the International Standard Classification of Education (ISCED-1997) [31]. A high education level was defined as holding a college or university degree (ISCED levels 5 and 6), a medium level as 12–13 years of education (ISCED levels 3 and 4), and a low level as nine years of education or less (ISCED levels 0, 1 and 2). Parents’ education level was defined by the highest level of the two parents if the child lived with both parents or in joint custody, and by the education level of the parent that the child lived with when it was only one parent.

*Family structure* was categorized into four groups as suggested by Bergström et al. [19]. These groups captured whether the child during the past year had lived with: two parents; in joint custody, i.e. alternating between separated or divorced parents; mainly with one parent, i.e. lived with one parent for most of the week; or with one parent and another adult.

### Statistical methods

Logistic regression was used to study the association between each of the five HRQoL dimensions and various potential explanatory factors (study year, municipality population size, migration status, parents’ occupation, sex, parents’ education level, and family structure). The analyses were also performed based on fathers’ or mothers’ education separately. However, the results are not presented here as the analysis showed similar findings. Odds ratios (OR) with 95% confidence interval (CI) were reported. Model fit was evaluated with Wald’s test [32], and was tested for possible multicollinearity with the variance inflation factor (VIF). Results were assessed through stratifications by all used factors to verify that no obvious pattern, such as effect modification, existed. Stata 13 was used for the statistical analyses (StataCorp LP, College Station, Texas, USA). A statistically significant level of 0.05 was applied.

## Results

Characteristics of the study population and prevalence of reported HRQoL problems are presented in Table 1. About half of the participants were girls, and around 80 % had a Swedish origin. Skilled non-manual workers were the most common parents’ occupation and nearly 50% had at least one parent with a high education level. Over half of the parents who were registered as students were from countries considered as low-income countries. The most commonly reported problems were pain followed by mood problems, while the prevalence of problems related to self-care were very low and therefore results from this dimension were not further analyzed.

**Table 1:** Characteristics and prevalence of children reporting problems in health-related quality of life as measured by EQ-5D-Y among 12-year-olds in Sweden. ETICS study 2005, 2009

Characteristic	*n*	(%)
Study year		
2005	6449	(58.6)
2009	4560	(41.4)
Municipality size		
> 50,000 inhabitants	7505	(68.2)
10,000 to 50,000	2943	(26.7)
< 10,000 inhabitants	561	(5.1)
Migration status		
Swedish origin	8516	(79.3)
One foreign parent	1045	(9.7)
Two foreign parents	768	(7.2)
Foreign origin	416	(3.9)
Parents’ occupation		
Skilled non-manual	4802	(46.6)
Unskilled non-manual	3205	(31.1)
Skilled manual	1399	(13.6)
Unskilled manual	793	(7.7)
Students	108	(1.1)
Sex		
Boys	5582	(50.7)
Girls	5427	(49.3)
Parents’ education		
High	5478	(51.0)
Medium	4762	(44.3)
Low	508	(4.7)
Family structure		
Both parents	8198	(76.6)
Joint custody	809	(7.6)
Mainly one parent	1084	(10.1)
Parent and another adult	618	(5.8)
HRQoL		
Mobility problems	310	(2.8)
Self-care problems	57	(0.5)
Problems in performing usual activities	243	(2.2)
Pain	2289	(20.8)
Mood problems	1413	(12.8)

Analyses of children’s questionnaires with missing parents’ responses showed somewhat higher prevalence of pain (21.9%) and mood problems (15.5%).

### Comparisons between 2005 and 2009

Children in the second cohort (2009) had OR’s of 1.2 in pain and 1.35 in mood problems, as compared to the earlier cohort (2005), both being statistically significant differences. However, there were no statistically significant differences between the two cohorts in the other HRQoL dimensions (Table 2).

**Table 2:** Disparities among 12-year-olds in health-related quality of life between different study years and sociodemographic subgroups

Characteristic		Mobility problems		Problems in performing usual activities		Pain		Mood problems	
	n	Crude OR^a^ OR (CI)	Adj. OR^b^ (CI)^a^	Crude OR (CI)	Adj. OR	Crude OR (CI)	Adj. OR	Crude OR (CI)	Adj. OR
Study year									
2005 (ref)^c^	6449	1	1	1	1	1	1	1	1
2009	4560	1.04 (0.8–1.3)	1.07 (0.8–1.4)	0.94 (0.7–1.2)	1.01 (0.8–1.3)	**1.17 (1.1–1.3)**	**1.20 (1.1–1.3)**	**1.38 (1.2–1.5)**	**1.35 (1.2–1.5)**
Municipality size									
> 50,000 inhabitants (ref)	7505	1	1	1	1	1	1	1	1
10,000 to 50,000	2943	1.15 (0.9–1.5)	1.25 (0.9–1.6)	0.95 (0.7–1.3)	1.00 (0.7–1.4)	0.97 (0.9–1.1)	0.96 (0.9–1.1)	0.99 (0.9–1.1)	0.97 (0.9–1.1)
< 10,000 inhabitants	561	**1.58 (1.0–2.5)**	1.47 (0.9–2.4)	**2.16 (1.4–3.3)**	**2.46 (1.6–3.8)**	**1.29 (1.1–1.6)**	**1.29 (1.0–1.6)**	1.00 (0.8–1.3)	1.06 (.80–1.4)
Migration status									
Swedish origin (ref)	8516	1	1	1	1	1	1	1	1
One foreign parent	1045	1.16 (0.8–1.7)	1.18 (0.8–1.7)	0.70 (0.4–1.2)	0.80 (0.5–1.3)	1.04 (0.9–1.2)	1.00 (0.9–1.2)	**1.27 (1.1–1.5)**	**1.25 (1.0–1.5)**
Two foreign parents	768	1.00 (0.6–1.6)	0.85 (0.5–1.5)	0.90 (0.5–1.5)	0.90 (0.5–1.7)	0.91 (0.8–1.1)	0.89 (0.7–1.1)	0.89 (0.7–1.1)	0.95 (0.7–1.2)
Foreign origin	416	1.45 (0.9–2.4)	1.61 (0.9–3.0)	0.94 (0.5–1.9)	0.99 (0.4–2.3)	0.79 (0.6–1.0)	0.75 (0.6–1.0)	0.95 (0.7–1.3)	1.00 (0.7–1.4)
Parents’ occupation									
Skilled non-manual (ref)	4802	1	1	1	1	1	1	1	1
Unskilled non-manual	3205	1.02 (0.8–1.3)	1.00 (0.7–1.3)	0.91 (0.7–1.2)	0.87 (0.6–1.2)	1.09 (1.0–1.2)	1.10 (1.0–1.2)	0.92 (0.8–1.1)	0.94 (0.8–1.1)
Skilled manual	1399	1.46 (1.0–2.0)	1.20 (0.8–1.8)	1.32 (0.9–1.9)	1.14 (0.7–1.8)	1.06 (0.9–1.2)	1.05 (0.9–1.2)	0.99 (0.8–1.2)	1.04 (0.8–1.3)
Unskilled manual	793	1.60 (1.1–2.4)	1.43 (0.9–2.3)	1.33 (0.8–2.1)	1.28 (0.8–2.2)	1.18 (1.0–1.4)	1.18 (1.0–1.4)	1.04 (0.8–1.3)	1.08 (0.8–1.4)
Student	108	2.28 (1.0–5.3)	2.20 (0.9–5.5)	0.43 (0.1–3.1)	0.51 (0.1–3.9)	1.31 (0.8–2.0)	1.61 (1.0–2.6)	1.60 (1.0–2.6)	1.70 (1.0–2.9)
Sex									
Boys (ref)	5582	1	1	1	1	1	1	1	1
Girls	5427	1.01 (0.8–1.3)	1.01 (0.7–1.3)	1.09 (0.8–1.4)	1.09 (0.8–1.4)	**1.22 (1.1–1.3)**	**1.20 (1.1–1.3)**	**2.65 (2.4–3.0)**	**2.57 (2.3–2.9)**
Parents’ education level									
High (ref)	5478	1	1	1	1	1	1	1	1
Medium	4762	**1.40 (1.1–1.8)**	1.28 (1.0–1.7)	1.08 (0.8–1.4)	0.91 (0.7–1.3)	1.02 (0.9–1.1)	0.96 (0.9–1.1)	0.89 (0.8–1.0)	**0.84 (0.7–0.96)**
Low	508	1.48 (0.9–2.5)	0.98 (0.5–1.9)	0.83 (0.4–1.6)	0.77 (0.4–1.7)	0.97 (0.8–1.2)	0.92 (0.7–1.2)	0.76 (0.6–1.0)	**0.65 (0.5–0.92)**
Family structure									
Both parents (ref)	8198	1	1	1	1	1	1	1	1
Joint custody	809	1.04 (0.7–1.6)	1.11 (0.7–1.7)	1.17 (0.7–1.9)	1.13 (0.7–1.9)	1.17 (1.0–1.4)	1.14 (1.0–1.4)	**1.39 (1.1–1.7)**	**1.36 (1.1–1.7)**
Mainly one parent	1084	1.17 (0.8–1.7)	1.08 (0.7–1.6)	1.20 (0.8–1.8)	1.19 (0.8–1.9)	**1.31 (1.1–1.5)**	**1.31 (1.1–1.5)**	**1.51 (1.3–1.8)**	**1.57 (1.3–1.9)**
Parent and another adult	618	1.49 (1.0–2.3)	1.20 (0.8–1.9)	**1.71 (1.1–2.7)**	**1.66 (1.0–2.7)**	1.20 (1.0–1.4)	1.20 (1.0–1.5)	**1.32 (1.0–1.7)**	**1.33 (1.0–1.7)**

^a^Odds ratio with 95% confidence interval in parenthesis; ^b^ Odds ratio adjusted for study year, municipality population size, migration status, parents’ occupation, sex, parents’ education level, and family structure; ^c^ Ref = Reference group of each characteristic. Statistically significant results in **bold**. Health-related quality of life is measured by EQ-5D-Y (The prevalence of problems related to self-care were very low and therefore not presented here). ETICS study 2005, 2009

### Comparison between sociodemographic groups

Overall, there were few observed disparities in HRQoL between sociodemographic subgroups (Table 2). Detailed numbers and prevalence of children who reported HRQoL problems according to different sociodemographic groups are shown in Additional file 1. Municipality population size was associated with two of the studied HRQoL dimensions. Children who lived in small compared to large municipalities had statistically significantly higher odds in two of the dimensions; OR’s were 2.46 in problems performing usual activities and 1.29 in pain. Regarding migration status, the only statistically significant difference was among children with one foreign parent who had an OR of 1.3 in mood problems compared with children of Swedish origin. For parents’ occupation, there were no statistically significant differences between groups (Table 2).

Compared to boys, girls had OR’s of 1.2 in pain and 2.57 in mood problems (Table 2). Only in mood problems there were statistically significant differences based on parents’ education levels. Children of parents with low or medium levels of education had OR’s of 0.84 respectively 0.65 in mood problems compared to children of parents with high education levels.

Family structure was the factor associated with the most widespread statistically significant HRQoL disparities (Table 2). Compared to children living with two parents, children living with one parent and one other adult had an OR of 1.66 in problems performing daily activities, and the OR of pain was 1.3 in children living mainly with only one parent. Finally, compared to children living with two parents, children in all other family constellations studied had OR’s of 1.33 to 1.57 in mood problems.

### Comparison between boys and girls

**Fig. 1:**
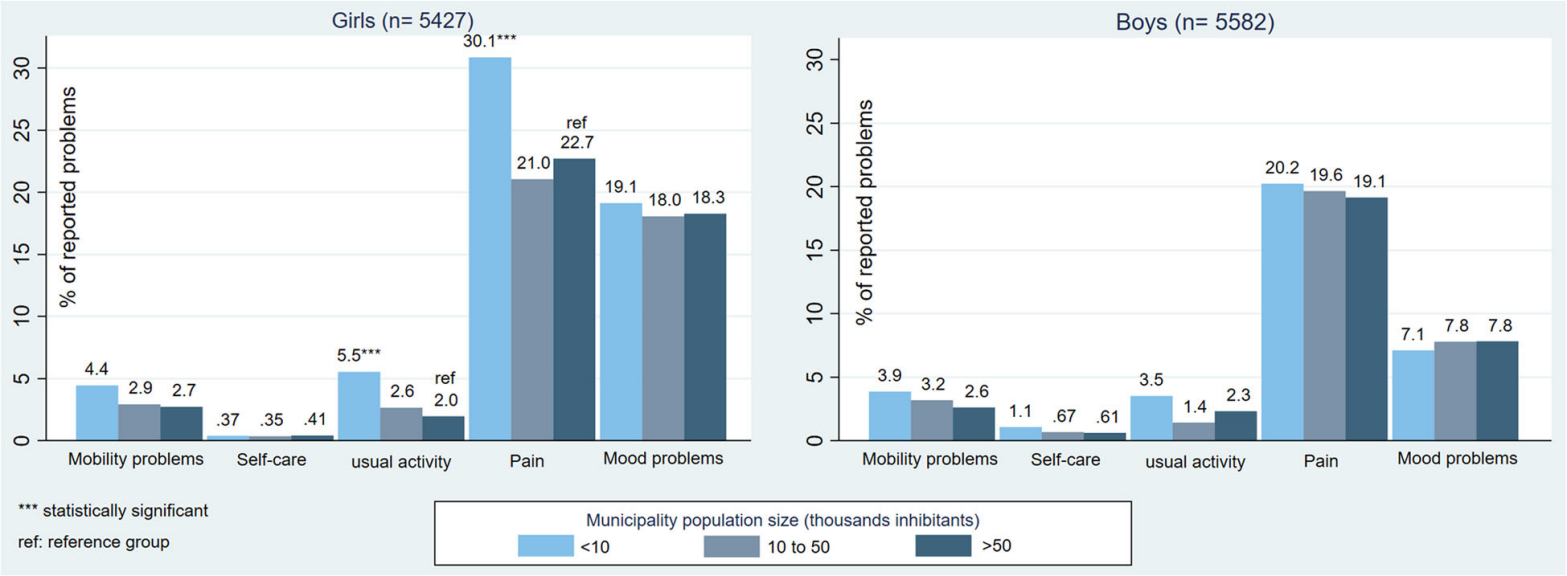
Comparison of health-related quality of life between boys and girls living in municipalities with different population sizes. ETICS study 2005, 2009

After stratifying by sex there were few disparities in HRQoL in girls but even fewer in boys (Table 3). For comparisons between cohorts, pain was only statistically significant among girls with an OR of 1.25 in the second cohort compared with the first cohort. Both boys and girls had a statistically significant increase in mood problems in the second cohort (OR’s of 1.37 in girls and 1.34 in boys) compared to the first cohort. Living in small municipalities showed statistically significant disparities only among girls. Girls living in small municipalities reported more than three times higher odds of problems performing usual activities and had an OR of 1.51 in pain compared to those who lived in large municipalities. While, no statistically significant disparities were seen among boys in any of the measured HRQoL dimensions. Percentages of reported HRQoL problems in different municipality population sizes among boys and girls are shown in Fig. 1.

**Table 3:** Disparities among 12-year-old boys and girls in health-related quality of life between different study years and sociodemographic subgroups

Characteristic	Mobility problems		Problems in performing usual activities		Pain		Mood problems	
	Boys	Girls	Boys	Girls	Boys	Girls	Boys	Girls
	Adj. OR^a^	Adj. OR	Adj. OR	Adj. OR	Adj. OR	Adj. OR	Adj. OR	Adj. OR
Study year								
2005 (ref)^b^	1	1	1	1	1	1	1	1
2009	0.92 (0.6–1.3)	1.24 (0.9–1.8)	1.00 (0.7–1.5)	1.03 (0.7–1.5)	1.15 (1.0–1.3)	**1.25 (1.1–1.4)**	**1.37 (1.1–1.7)**	**1.34 (1.2–1.6)**
Municipality size								
> 50,000 inhabitants (ref)	1	1	1	1	1	1	1	1
10,000 to 50,000	1.39 (1.0–2.0)	1.09 (0.7–1.6)	0.66 (0.4–1.1)	1.44 (0.9–2.2)	1.02 (0.9–1.2)	0.90 (0.8–1.1)	0.98 (0.8–1.2)	0.97 (0.8–1.2)
< 10,000 inhabitants	1.53 (0.8–3.0)	1.45 (0.7–2.8)	1.59 (0.8–3.1)	**3.77 (2.1–6.8)**	1.07 (0.8–1.5)	**1.51 (1.1–2.0)**	0.99 (0.6–1.6)	1.09 (0.8–1.5)
Migration status								
Swedish origin (ref)	1	1	1	1	1	1	1	1
One foreign parent	1.20 (0.7–2.1)	1.16 (0.7–2.1)	0.83 (0.4–1.7)	0.78 (0.4–1.6)	0.97 (0.8–1.2)	1.04 (0.8–1.3)	1.32 (1.0–1.8)	1.21 (0.9–1.5)
Two foreign parents	0.46 (0.2–1.3)	1.27 (0.7–2.4)	0.82 (0.4–1.9)	1.01 (0.4–2.4)	0.78 (0.6–1.1)	1.02 (0.8–1.4)	1.19 (0.8–1.8)	0.84 (0.6–1.2)
Foreign origin	1.75 (0.7–4.1)	1.39 (0.5–3.5)	0.65 (0.2–2.8)	1.38 (0.5–3.9)	0.99 (0.6–1.5)	**0.59 (0.4–0.9)**	1.27 (0.7–2.3)	0.87 (0.6–1.4)
Parents’ occupation								
Skilled non-manual (ref)	1	1	1	1	1	1	1	1
Unskilled non-manual	1.09 (0.7–1.6)	0.91 (0.6–1.4)	1.11 (0.7–1.8)	0.67 (0.4–1.1)	1.10 (0.9–1.3)	1.10 (0.9–1.3)	0.82 (0.6–1.1)	1.00 (0.8–1.2)
Skilled manual	0.83 (0.5–1.5)	1.58 (0.9–2.6)	1.39 (0.8–2.6)	0.94 (0.5–1.8)	1.03 (0.8–1.3)	1.07 (0.8–1.4)	1.15 (0.8–1.6)	0.99 (0.8–1.3)
Unskilled manual	1.18 (0.6–2.4)	1.70 (0.9–3.2)	1.35 (0.6–2.9)	1.22 (0.6–2.6)	1.21 (0.9–1.6)	1.17 (0.9–1.6)	0.96 (0.6–1.5)	1.15 (0.8–1.6)
Student	**4.29 (1.5–11.)**	n/a^c^	1.52 (0.2–12.)	n/a	**2.38 (1.2–4.6)**	1.01 (0.5–2.1)	1.32 (0.5–3.5)	**1.99 (1.0–3.9)**
Parents’ education level								
High (ref)	1	1	1	1	1	1	1	1
Medium	1.28 (0.9–1.9)	1.30 (0.9–1.9)	1.30 (0.8–2.0)	0.64 (0.4–1.0)	0.99 (0.8–1.2)	0.94 (0.8–1.1)	**0.77 (0.6–1.0)**	0.87 (0.7–1.0)
Low	1.23 (0.5–2.9)	0.80 (0.3–2.1)	0.75 (0.2–2.6)	0.74 (0.3–2.0)	0.78 (0.5–1.2)	1.04 (0.7–1.5)	0.57 (0.3–1.1)	0.68 (0.4–1.0)
Family structure								
Both parents (ref)	1	1	1	1	1	1	1	1
Joint custody	1.25 (0.7–2.3)	0.97 (0.5–1.9)	0.68 (0.3–1.6)	1.78 (0.9–3.4)	1.10 (0.8–1.4)	1.19 (0.9–1.5)	1.27 (0.9–1.8)	**1.42 (1.1–1.9)**
Mainly one parent	0.99 (0.5–1.8)	1.18 (0.7–2.0)	0.83 (0.4–1.7)	1.62 (0.9–3.0)	1.23 (1.0–1.6)	**1.40 (1.1–1.7)**	**1.47 (1.0–2.0)**	**1.63 (1.3–2.1)**
Parent and another adult	0.88 (0.4–1.8)	1.61 (0.9–3.0)	1.17 (0.6–2.5)	**2.40 (1.2–4.6)**	1.20 (0.9–1.6)	1.19 (0.9–1.6)	1.40 (0.9–2.1)	1.28 (0.9–1.8)

^a^Odds ratio adjusted for study year, municipality population size, migration status, parents’ occupation, sex, parents’ education level, and family structure; ^b^ Ref = Reference group of each characteristic. Statistically significant results in **bold**. ^c^ n/a = not applicable due to too few children with problems in the group. Health-related quality of life is measured by EQ-5D-Y (The prevalence of problems related to self-care were very low and therefore not presented here). ETICS study 2005, 2009

Girls with a foreign origin had statistically significant less perceived pain (OR of 0.59) compared to girls with a Swedish origin. Moreover, children of student parents were identified as a disadvantaged group in both boys and girls. Girls of student parents had a statistically significant double odds of mood problems compared to girls of skilled non-manual worker. While boys who had student parents had more than double as high odds in pain problems and four times higher odds in mobility problems compared to boys of skilled non-manual workers. On the other hand, the association between parents’ education and mood problems was only statistically significant among boys. Boys of parents with a medium level of education had an OR of 0.77 in mood problems compared to boys of parents with high education levels. Regarding family structure, girls living in joint custody had an OR of 1.4 in mood problems compared to girls living with two parents, while no statistically significant differences were detected among boys. However, girls who lived with one parent and another adult had more than double as high odds of problems performing usual activities compared to those living with two parents. Compared to those living with two parents, girls living mainly with one parent had OR’s of 1.6 in mood problems and 1.4 in pain while, boys living mainly with one parent had an OR of 1.5 in mood problems compared to those living with two parents. Contrary to non-stratified data, living with one parent and another adult did not show statistically significant results regarding mood problems.

Except for the statistically significant results when stratifying by sex, other stratification did not show noteworthy differences e.g., stratifications by study sites, municipalities, municipality population size, migration status, parents’ occupation, parents’ education level, or family structure.

## Discussion

In this study, we have shown that HRQoL problems related to pain and mood problems were higher in 2009 compared to 2005 among 12-year-olds in Sweden. Our study revealed few sociodemographic disparities in HRQoL in 12-year-olds and these disparities were mainly among girls. In general, girls reported more pain and mood problems than boys, however, there were no differences based on parents’ occupation. Of the five HRQoL dimensions, only one, mood problems, differed by parents’ education level where a higher parents’ education was unfavorable. Municipality size and family structure were associated with different levels of pain and abilities to perform usual activities among girls. In addition, children living with two parents had less mood problems than children living in other family constellations.

This study shows an increased prevalence of children reporting pain and mood problems in the more recent cohort of preteen children. Previous literature is conflicting. One study reported similar results between 2003 and 2011 among 12-year-olds in Northern Sweden when using parent-proxy EQ-5D measures [14]. However, based on the same type of data, another study found no upward time trend for pain or mood problems between 2003 and 2011 in a sample of preteen children representing the whole of Sweden [33]. Studies that used a scale of four or five levels of self-rated health, also reported no changes in self-perceived health over time [14, 33]. The possible reasons behind the health differences between 2005 and 2009 that we found in this study were not investigated. However, based on the results of other studies we speculate in the following explanations: the worsening of body image especially among adolescent girls [15], the increased feeling of pressure in schools [15], and the 2008 economic recession which had negative impact on child’s health in several high income countries [34]. Reasons behind these health differences between 2005 and 2009 should be further studied and future trends in self-rated HRQoL among preteen children should also continue to be reported.

This study reveals a negative relation between living in small municipalities and preteens’ HRQoL especially among girls. Girls living in small municipalities perceived more problems performing usual activities and pain. The literature about this relation between rurality and child’s health problems is conflicting. In one study, self-reported subjective health complaints, including pain and nervousity, were not related to residential area in children aged 11, 13 and 15 years [35]. While another study shows more problems related to pain and performing usual activities in preteen children living in rural area [36]. Together these findings indicate that the association between municipality size and health problems may vary depending on the age of the child. The better HRQoL in children living in a large size municipality may be partly explained by an expected greater access to neighborhood services in larger municipalities (sidewalks, parks, or playing areas), and increased feeling of safety and satisfaction [36]. Another explanation could be that preteen children who live in small municipalities feel more isolated and restricted and they perceive lower social capital due to daily environmental and social constraints [37]. Even though a stronger societal cohesion in these areas promotes wellbeing, it might promote exclusion and social control as well [37].

This study shows more mood problems among preteens of one foreign parent. Contrary, girls with foreign origin showed less pain. The few studies that have looked into this before, have shown inconsistent results; one study reported more subjective health complaints among girls of foreign origin. Another study showed that children from EU/OECD background experienced less psychosomatic symptoms than those of Swedish background while other migrants experienced same level of symptoms [22]. These inconsistent results might be due to different age group studied or to the heterogeneity of migrants and their migration motives in Sweden. More studies are needed to get a better understanding of HRQoL in children of foreign-born parent(s).

We did not find an association between parents’ occupation and preteens’ HRQoL except for children of student parents who reported more HRQoL problems. More than half the parents in this group held a low-income nationality, which gives the impression that this disadvantaged group consists mostly of migrant parents most probably attending Swedish language schools. Another Swedish study showed no association between mental problems and parents’ occupation among preteens as well [38].

Our results of more mood problems experienced among children of parents’ with a high education level is not in line with other European studies which suggest better health status among children of parents’ with a high education level [3, 23, 36], or a Swedish study among 12-year-olds which shows that those with lower parents’ education level generally have poorer HRQoL using parent-proxy EQ-5D [33]. The reason behind these health disparities is a topic of further research.

The association between family structure and HRQoL, reported in our study is in line with a previous study which shows better HRQoL among children living with two parents or in joint custody [19]. Our study highlights a greater susceptibility for girls who have more disparities in HRQoL related to family structure than boys.

### Methodological considerations

A strength in our study is that our results are based on a large sample size, corresponding to approximately 10% of the twelve-year-olds in Sweden, with a relatively high participation rate (60% of all invited participants were included in this study). Still, non-respondents might have higher levels of HRQoL problems. Furthermore, there were differences in HRQoL dimensions between our sample and children with missing parents’ responses. However, we expect these differences to at most marginally affect our associations. Moreover, the sample has been considered representative of children of the same age in Sweden according to previous studies based on geographical representation, healthcare consumption, unemployment rates and HRQoL [39, 40]. The high response rate and the similar methods in 2005 and 2009 support the generalization of our results to twelve-year-olds in Sweden. However, these results might not apply on other age groups or countries with different living standards than Sweden.

There are both a strength and a limitation in our use of EQ-5D-Y in this study. On the one hand, there is support from Swedish-based studies on the validity and reliability of this tool including test retest reliability and discriminative validity [25, 26]. On the other hand, the ceiling effect might have limited our analysis.

Even though geographical data were collected in the study, we did not consider using a multilevel analysis as initial analyses showed very little contribution to the models.

We apply logistic regression for four of the EQ-5D-Y domains and with stratified analyses by sex and estimates for the exposure variables, we present results from 184 tests. There is therefore a risk of spurious associations, and adjustments for multiple testing might be justifiable. However, we followed recommendations from methodologists [41, 42] and did not adjust for multiple testing as this might adventure the possibility to provide valid information about associations. Instead, we recommend that the associations provided are interpreted with care due to the multiple testing.

One recognized limitation of our study is that it does not include some relevant variables to children’s health such as social capital or neighborhood’s characteristics. Another major limitation in regard to migration analysis is that there is no differentiation between the reasons of migration e.g. labor, study or asylum seekers. This might influence the results by bringing heterogeneous groups in one category. Although the sample size of our study is large, the number of reported problems by some groups was relatively low in some of the EQ-5D dimensions especially after stratification.

## Conclusions

This study provides a valuable comparison of HRQoL status among twelve-year-olds in Sweden between 2005 and 2009. Our study indicates that HRQoL has worsened over this time period and that children of highly educated parents and those living in small municipalities have poorer health-related outcomes than others.

To study future trend, health outcomes among children in Sweden should continue to be reported periodically preferably with a self-rated HRQoL instrument. More efforts should be invested to increase the awareness of health disparities highlighted in this study especially regarding girls living in small municipalities and children of highly educated parents. Follow-up studies should be conducted to explain the reasons behind such disparities.

## Additional file

Additional file 1: “Prevalence of children reporting HRQoL.docx”. In the file is data presented in a table with the header: “Prevalence of children reporting health-related quality of life (HRQoL) problems by study year and sociodemographic characteristics in 12-year old children in Sweden (HRQoL measured by EQ-5D-Y). ETICS study 2005, 2009”. (DOCX 27 kb)

## Abbreviation

CI: Confidence interval
EQ-5D-Y: EuroQol 5 dimensions-youth
ETICS: Exploring the Iceberg of Celiacs in Sweden – screening study
HRQoL: Health-related quality of life
ISCED: International standard classification of education
OR: Odds ratio
SEI: Socioeconomic index
VIF: Variance inflation factor
