# Supplementary material for: Preteen children’s health related quality of life in Sweden: changes over time and disparities between different sociodemographic groups
Source: BMC Public Health. 2019 Jan 31;19:139. doi: 10.1186/s12889-019-6429-6 (PMC6357483; doi:10.1186/s12889-019-6429-6)
Supplement: Supplementary file 1 — “Prevalence of children reporting HRQoL.docx”. In the file is data presented in a table with the header: “Prevalence of children reporting health-related quality of life (HRQoL) problems by study year and sociodemographic characteristics in 12-year old children in Sweden (HRQoL measured by EQ-5D-Y). ETICS study 2005, 2009”. (DOCX 27 kb) [file 12889_2019_6429_MOESM1_ESM.docx]

# Additional file 1

Prevalence of children reporting health-related quality of life problems by study year and sociodemographic characteristics in 12-year old children in Sweden (HRQoL measured by the EQ-5D-Y). ETICS study 2005, 2009.

| **Characteristic** | n | Mobility  Problems  n (%) | | Self-care problems  n (%) | | Problems in performing usual activities  n (%) | | Pain  n (%) | | Mood problems  n (%) | |
| --- | --- | --- | --- | --- | --- | --- | --- | --- | --- | --- | --- |
| **Study year** |  |  |  |  |  |  |  |  |  |  |  |
| 2005 | 6449 | 179 | (2.8) | 31 | (0.5) | 146 | (2.3) | 1271 | (19.7) | 730 | (11.3) |
| 2009 | 4560 | 131 | (2.9) | 26 | (0.6) | 97 | (2.1) | 1018 | (22.3) | 683 | (15.0) |
|  |  |  |  |  |  |  |  |  |  |  |  |
| **Municipality size** |  |  |  |  |  |  |  |  |  |  |  |
| >50,000 inhabitants | 7505 | 198 | (2.6) | 38 | (0.5) | 159 | (2.1) | 1557 | (20.8) | 967 | (12.9) |
| 10,000 to 50,000 | 2943 | 89 | (3.0) | 15 | (0.5) | 59 | (2.0) | 592 | (20.1) | 374 | (12.7) |
| <10,000 inhabitants | 561 | 23 | (4.1) | 4 | (0.7) | 25 | (4.5) | 140 | (25.0) | 72 | (12.8) |
|  |  |  |  |  |  |  |  |  |  |  |  |
| **Migration status** |  |  |  |  |  |  |  |  |  |  |  |
| Swedish origin | 8516 | 232 | (2.7) | 42 | (0.5) | 196 | (2.3) | 1781 | (20.9) | 1072 | (12.6) |
| One foreign parent | 1045 | 33 | (3.2) | 5 | (0.5) | 17 | (1.6) | 226 | (21.6) | 161 | (15.4) |
| Two foreign parents | 768 | 21 | (2.7) | 4 | (0.5) | 16 | (2.1) | 150 | (19.5) | 88 | (11.5) |
| Foreign origin | 416 | 16 | (3.9) | 4 | (1.0) | 9 | (2.2) | 72 | (17.3) | 50 | (12.0) |
|  |  |  |  |  |  |  |  |  |  |  |  |
| **Parents’ occupation** |  |  |  |  |  |  |  |  |  |  |  |
| Skilled non-manual | 4802 | 121 | (2.5) | 18 | (0.4) | 102 | (2.1) | 967 | (20.1) | 633 | (13.2) |
| Unskilled non-manual | 3205 | 82 | (2.6) | 14 | (0.4) | 62 | (1.9) | 689 | (21.5) | 392 | (12.2) |
| Skilled manual | 1399 | 51 | (3.7) | 13 | (0.9) | 39 | (2.8) | 296 | (21.2) | 183 | (13.1) |
| Unskilled manual | 793 | 31 | (3.9) | 4 | (0.5) | 22 | (2.8) | 180 | (22.7) | 107 | (13.5) |
| Students | 108 | 6 | (5.6) | 1 | (0.9) | 1 | (0.9) | 27 | (25.0) | 21 | (19.4) |
|  |  |  |  |  |  |  |  |  |  |  |  |
| **Sex** |  |  |  |  |  |  |  |  |  |  |  |
| Boys | 5582 | 156 | (2.8) | 36 | (0.6) | 118 | (2.1) | 1067 | (19.1) | 429 | (7.7) |
| Girls | 5427 | 154 | (2.8) | 21 | (0.4) | 125 | (2.3) | 1222 | (22.5) | 984 | (18.1) |
|  |  |  |  |  |  |  |  |  |  |  |  |
| **Parents’ education level** |  |  |  |  |  |  |  |  |  |  |  |
| High | 5478 | 127 | (2.3) | 25 | (0.5) | 117 | (2.1) | 1137 | (20.8) | 738 | (13.5) |
| Medium | 4762 | 153 | (3.2) | 29 | (0.6) | 109 | (2.3) | 1001 | (21.0) | 580 | (12.2) |
| Low | 508 | 17 | (3.4) | 2 | (0.4) | 9 | (1.8) | 102 | (20.1) | 54 | (10.6) |
|  |  |  |  |  |  |  |  |  |  |  |  |
| **Family structure** |  |  |  |  |  |  |  |  |  |  |  |
| Both parents | 8198 | 216 | (2,6) | 43 | (0.5) | 165 | (2.0) | 1633 | (19.9) | 969 | (11.8) |
| Joint custody | 809 | 22 | (2.7) | 2 | (0.3) | 19 | (2.4) | 183 | (22.6) | 126 | (15.6) |
| Mainly one parent | 1084 | 33 | (3.0) | 7 | (0.7) | 26 | (2.4) | 266 | (24.5) | 182 | (16.8) |
| Parent and another adult | 618 | 24 | (3.9) | 4 | (0.7) | 21 | (3.4) | 142 | (23.0) | 93 | (15.1) |
